# Neuroendocrine carcinoma of the common bile duct associated with congenital bile duct dilatation: a case report

**DOI:** 10.1186/s12876-021-01777-7

**Published:** 2021-06-12

**Authors:** Yoshitaka Kiya, Yuichi Nagakawa, Chie Takishita, Hiroaki Osakabe, Hitoe Nishino, Masanori Akashi, Hiroshi Yamaguchi, Toshitaka Nagao, Ryo Oono, Kenji Katsumata, Akihiko Tsuchida

**Affiliations:** 1 Department of Gastrointestinal and Pediatric Surgery, Tokyo Medical University, 6-7-1 Nishi-shinjuku, Shinjuku- ku, Tokyo 160-0023 Japan; 2 Department of Surgery, Kurume University School of Medicine, 67 Asahicho, 830-0011 Kurume, Fukuoka Japan; 3 Department of Pathology, Saitama Medical University, 38 Morohongo, Moroyama-machi, Iruma-gun, Saitama, 350-0495 Japan; 4 Department of Anatomical Pathology, Tokyo Medical University, 6-7-1 Nishi-shinjuku, Shinjuku-ku, Tokyo 160- 0023 Japan; 5 Department of Digestive Surgery, Nitobe Memorial Nakano General Hospital, 4-59-16 Chuo, Nakano-ku, Tokyo 164-8607 Japan

**Keywords:** Neuroendocrine carcinoma, Congenital bile duct dilatation, Cholangiocarcinoma, Large cell neuroendocrine carcinoma, Adjuvant chemotherapy, Case report

## Abstract

**Background:**

Cholangiocarcinoma is frequently observed in patients with congenital bile duct dilatation (CBDD). Most cholangiocarcinomas are adenocarcinomas. Other types, especially neuroendocrine carcinomas (NECs), are rare. To the best of our knowledge, this is the third reported case of an NEC of the common bile duct associated with CBDD and the first to receive adjuvant chemotherapy for advanced disease.

**Case presentation:**

A 29-year-old woman presented with upper abdominal pain. Preoperative imaging indicated marked dilatation of the common bile duct and a tumor in the middle portion of the common bile duct. She was suspected of having distal cholangiocarcinoma associated with CBDD and underwent pylorus-preserving pancreaticoduodenectomy. Pathological and immunohistological findings led to a final diagnosis of large-cell NEC (pT3aN1M0 pStageIIB). The postoperative course was uneventful, and she was administered cisplatin and irinotecan every 4 weeks (four cycles) as adjuvant chemotherapy. She has remained recurrence-free for 16 months.

**Conclusions:**

NEC might be a differential diagnosis in cases of cholangial tumor associated with congenital bile duct dilatation. This presentation is rare and valuable, and to establish better treatment for NEC, further reports are necessary.

## Background

Congenital bile duct dilatation (CBDD) is found in approximately one in 100,000 to 150,000 people, and is frequently observed in Asian patients, including those of Japanese origin [1]. In Japanese patients with CBDD, the incidence of cholangiocarcinoma is approximately 15 %. The incidence of cholangiocarcinoma after extrahepatic bile duct excision is 0.7 %, which is approximately 200 times higher than the incidence of cholangiocarcinoma in the general population in Japan [2, 3]. Cholangiocarcinomas frequently develop in patients with CBDD and are usually adenocarcinomas; other types, especially neuroendocrine carcinomas (NEC), are rare [4].

We present a rare case of an NEC of the common bile duct associated with CBDD. To the best of our knowledge, this is the third case of an NEC of the common bile duct associated with CBDD to be reported [5, 6].

## Case presentation

A 29-year-old Japanese woman, with a history of cholecystitis at the age of 15 years and no relevant family history, presented to a hospital complaining of upper abdominal pain, which began to worsen one month ago.

The patient’s vital signs were stable. Physical examination revealed slight upper abdominal tenderness, without jaundice, and a positive Murphy’s sign and Blumberg’s sign.

Blood analysis revealed elevated levels of aspartate aminotransaminase, 309 IU/L (normal, 8–40 IU/L); alanine aminophosphatase, 511 IU/L (normal, 5–35 IU/L); γ-glutamyl transpeptidase, 614 IU/L (normal, 7–50 IU/L); alkaline phosphatase, 822 IU/L (normal, 100–340 IU/L); and carcinoembryonic antigen, 79.7 ng/mL (normal, 0–4.9 ng/mL). All other laboratory data were within normal limits. The levels of hepatobiliary enzymes and tumor markers were elevated, which implied that the patient might have developed cholangitis due to a cholangial carcinoma.

**Fig. 1:**
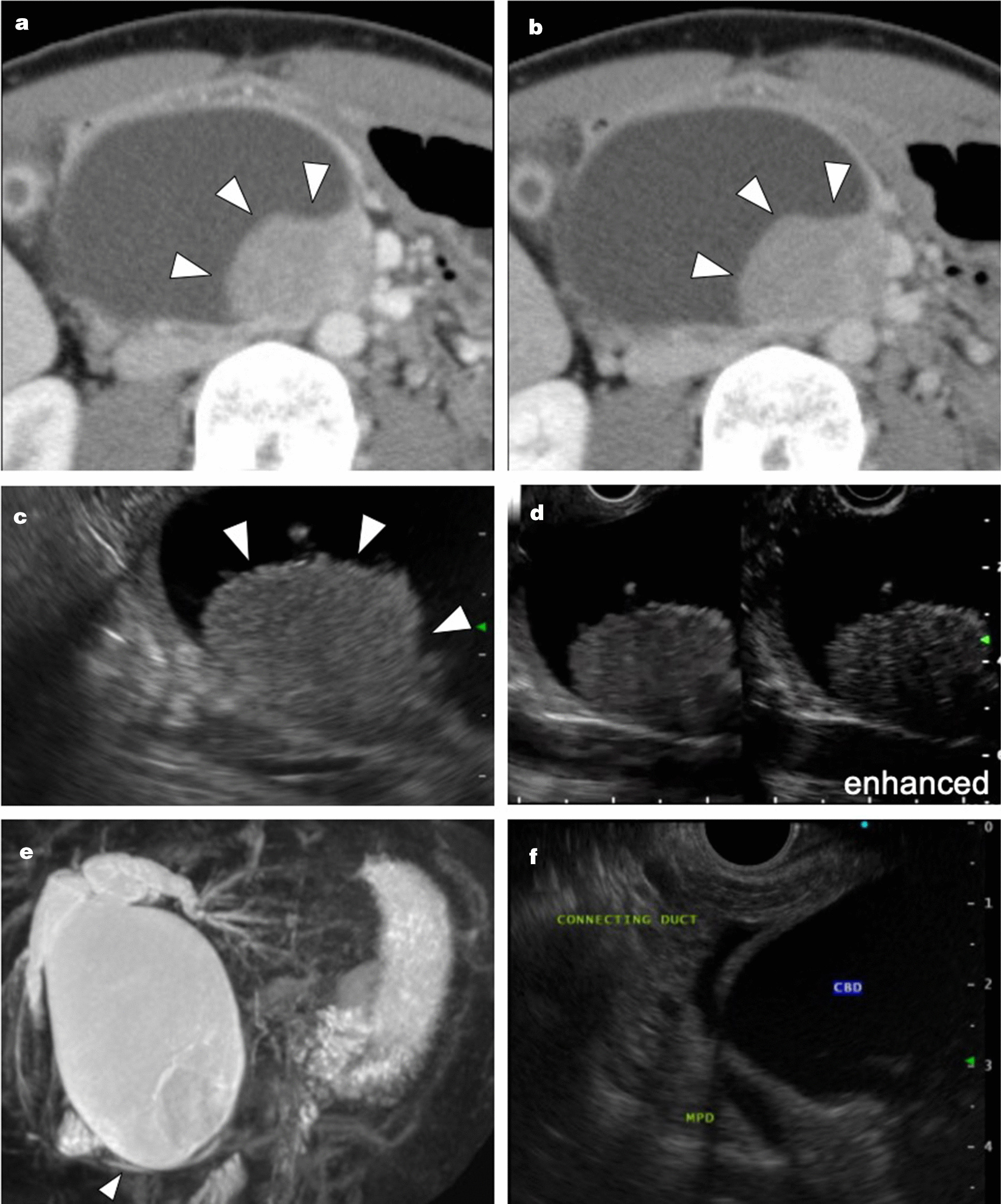
Preoperative imaging. **a**, **b** Arterial phase and delayed phase of abdominal dynamic contrast-enhanced computed tomography. These images show a tumor (arrowhead) in the middle portion of the common bile duct and dilatation of the common bile duct and the intrahepatic bile ducts bilaterally; the dilatation is diagnosed as Todani type IV-A. The tumor has enhanced iso-density at the arterial phase, and contrast effect was prolonged to the delayed phase. **c** Endoscopic ultrasonography shows a tumor (arrowhead) in the cystic dilated common bile duct. **d** Contrast-enhanced endoscopic ultrasonography shows the uptake of the contrast agent into the tumor. **e**, **f** Magnetic resonance cholangiopancreatography and endoscopic ultrasonography show one centimeter of the common channel (arrowhead), which continued from the duodenal papilla

Abdominal dynamic contrast-enhanced computed tomography (CECT) showed dilatation of the common bile duct and the bilateral intrahepatic bile ducts (which was diagnosed as Todani type IV-A) and a tumor in the middle portion of the common bile duct (Fig. 1a, b). Endoscopic ultrasonography and magnetic resonance cholangiopancreatography showed a tumor in the cystic dilated common bile duct (Fig. 1c, 1d); additionally, one centimeter of the common channel continued from the duodenal papilla (Fig. 1e, f), and no evidence of choledocholithiasis was observed. Considering all factors, since cholangial carcinoma was highly suspected, endoscopic retrograde cholangiopancreatography and peroral cholangioscopy were not performed to prevent pancreatitis.

The patient was suspected of having distal cholangiocarcinoma (cT2N0M0 cStageIB) associated with CBDD and underwent pylorus-preserving pancreaticoduodenectomy with modified Child reconstruction. Intraoperative observation of the abdominal cavity did not reveal any feature of tumor dissemination or liver metastasis. The surgery was performed as scheduled.

**Fig. 2:**
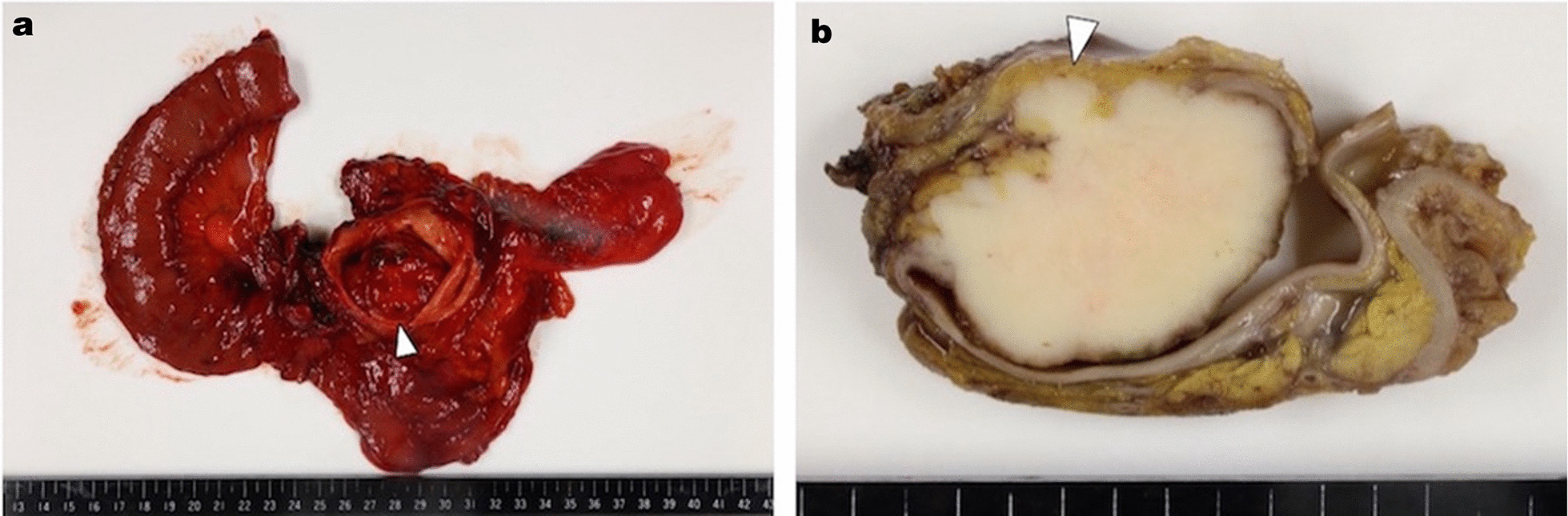
Macroscopic finding. **a** A 50-mm sessile irregular lesion (arrowhead) protruding into the lower common bile duct is observed. **b** Gross description of the short axis of the common bile duct. Tumor invasion into the pancreas (arrowhead)

**Fig. 3:**
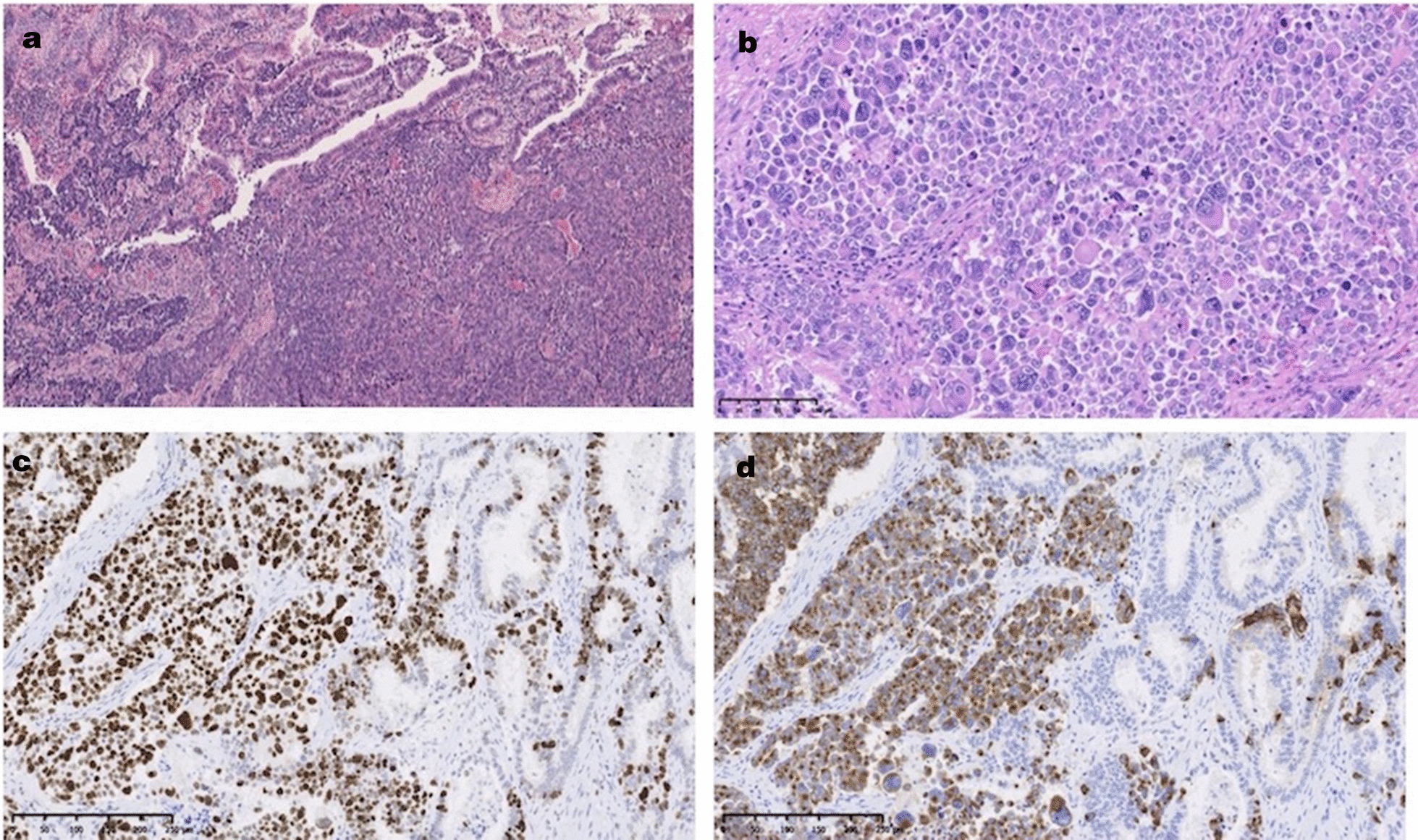
Microscopic findings; hematoxylin and eosin stain and immunohistochemical stain. **a** The transition area from the adenocarcinoma with a tubulopapillary structure (left upper part) to the neuroendocrine carcinoma (NEC) with a solid structure (right lower part). Hematoxylin and eosin stain (H&E, ×60). **b** The area of NEC consisting of large tumor cells with irregular-shaped hyperchromatic nuclei (H&E, ×200). **c** Ki-67 labeling index was over 90 % solid component (×100). **d** Tumor cells are positive for chromogranin-A in the solid component, thereby revealing neuroendocrine differentiation (×100)

The final diagnosis was large-cell NEC (pT3aN1M0 pStageIIB), and the resection margin was negative. Macroscopic findings showed a 50-mm sessile irregular lesion protruding into the markedly dilated lower common bile duct (Fig. 2a, b). Pathological findings showed solid proliferation of large, atypical cells accompanied by an adenocarcinomatous component (less than 5 %); i.e. the transition area of these component (Fig. 3a), and large tumor cells with irregular-shaped hyperchromatic nuclei (Fig. 3b). Metastasis was detected in one of the 31 lymph nodes tested. Immunohistochemical studies revealed that the large, atypical cells were positive for AE1/AE3, E-cadherin, CD56, chromogranin A, and synaptophysin. The Ki-67 labeling index was over 90 % in the solid component (Fig. 3c, d).

The postoperative course was uneventful, and she was discharged on the 14th postoperative day. This was a case of an advanced NEC, and she was scheduled to receive adjuvant chemotherapy to prevent recurrence. However, the standard regimen of adjuvant chemotherapy for neuroendocrine cholangiocarcinoma has not yet been established; therefore, we selected a mixed regimen of cisplatin and irinotecan according to the National Comprehensive Cancer Network (NCCN) guidelines for small cell carcinomas of the lung. The patient received cisplatin at a dose of 60 mg/m^2^ on the 1st day and irinotecan at 60 mg/m^2^ on the 1st, 8th, and 15th days. This regimen was repeated every 4 weeks for four cycles as adjuvant chemotherapy. She has been recurrence-free for over 16 months.

## Discussion and conclusions

The most common form of cholangiocarcinoma associated with CBDD is adenocarcinoma; NECs are rare, and only two cases have been reported in the English literature (Table 1). Pathological diagnoses of the previously reported cases were of early well-differentiated NECs [5, 6]. This is the third case of an NEC of the common bile duct associated with CBDD and the first case to receive adjuvant chemotherapy for advanced disease.

**Table 1:** Case reports of neuroendocrine carcinomas of the common bile duct associated with congenital bile duct dilatation

Case	Author	Age/sex	Todani type	Diagnosis	Treatment	Prognosis
1	Tonnhofer	6/F	I	Well-differentiated neuroendocine carcinoma	Total extrahepatic bile duct resection	no evidence of recurrence 2y
2	Takahashi	28/F	IV-A	Well-differentiated neuroendocine carcinoma	Total extrahepatic bile duct resection Pancreaticoduodenectomy	no evidence of recurrence 3y
3	Present case	29/F	IV-A	Poorly-differentiated neuroendocine carcinoma	Pylorus-preserving pancreaticoduodenectomy Adjuvant chemotherapy	no evidence of recurrence 16mo

CBDD is observed more often in female patients than in male patients (male-to-female ratio, 1:3). Similarly, CBDD is common in Asian patients. Todani et al., categorized CBDD into five main types, and almost all CBDDs of types Ia, Ic, and IV-A are associated with pancreaticobiliary maljunction [2].

A proposed etiology of bile duct dilatation is an increase in the bile duct pressure of the narrow segment of the lower bile duct accompanied by pancreaticobiliary maljunction while the bile duct wall is immature, thereby resulting in the dilation of the bile duct. Another hypothesis is that the primitive common bile duct proliferates asymmetrically in the early embryonic period; this implies that insufficient epithelial proliferation in the lower bile duct and excessive proliferation in the upper bile duct lead to stenosis of the lower bile duct and dilatation of the upper bile duct [2].

Pancreaticobiliary maljunction causes mixing and regurgitation of bile and pancreatic juices, which stagnate in the gallbladder and the bile duct, especially in the dilated common bile duct. This increases the cytotoxic potential for damage to the biliary epithelium under conditions of infection, inflammation, biliary stasis, decreased trypsin inhibitor concentrations, and the presence of enterokinase [5, 7, 8]. During repair, multiple alterations of oncogenes and tumor suppressor genes occur, and this could lead to the development of carcinomas through multistage interactions. Hyperplasia of the biliary or gallbladder epithelium is a characteristic of pancreaticobiliary maljunction; moreover, mutations of the *K-ras* and *p53* genes and overexpression of the p53 protein are observed in malignant and benign lesions of the biliary tract in patients with pancreaticobiliary maljunction. These changes are referred to as the “hyperplasia-dysplasia-carcinoma sequence” [9, 10].

In many anatomical sites, neoplasms that exhibit both neuroendocrine and non-neuroendocrine elements exist, which can be present as morphologically distinct or more intimately intermixed cell populations. The neuroendocrine elements of these “mixed” or “combined” neoplasms are most commonly NECs; the non-neuroendocrine components can be glandular, squamous, or of other lineages [11].

**Fig. 4:**
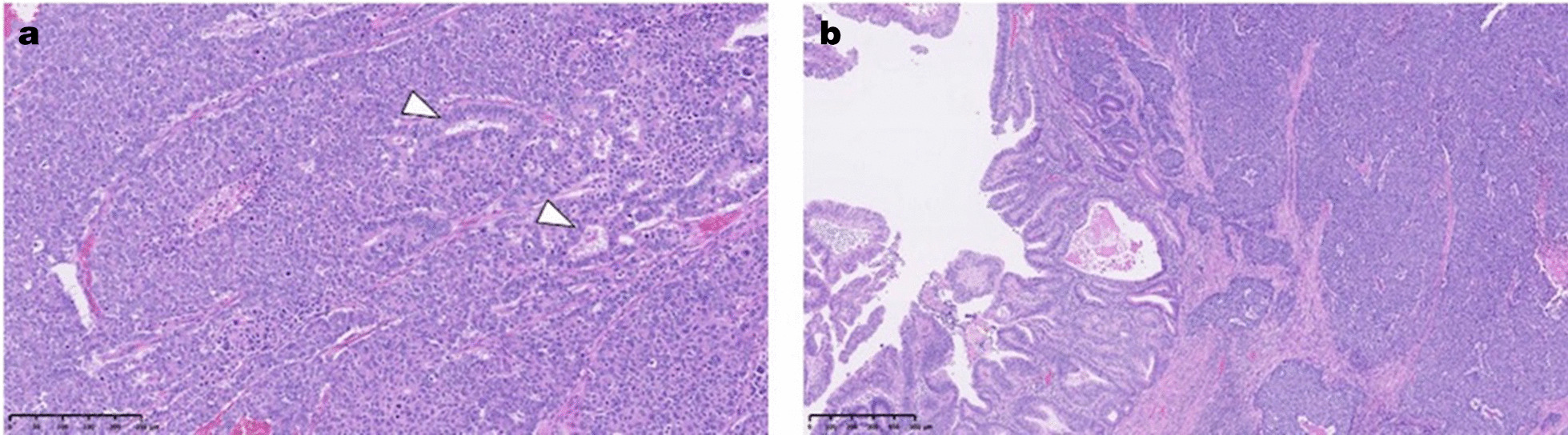
Microscopic findings; neuroendocrine components and adenocarcinomatous components. **a** Neuroendocrine components occupy 90 % or more of the tumor invasive area, and a few adenocarcinoma components are observed (arrowhead) (H&E, ×100). **b** Early adenocarcinoma components spread on the mucosa of the tumor surface (H&E, ×40)

In our case, neuroendocrine components occupied 90 % or more of the tumor’s invasive area, and a few adenocarcinomatous components were observed (Fig. 4a). In contrast, early adenocarcinomatous components were observed on the mucosa of the tumor surface (Fig. 4b). This suggests that hyperplasia and dysplasia due to chronic inflammation of the bile duct mucosa initially result in adenocarcinomas, which subsequently transform to NECs (Fig. 3a); however, in our case, hyperplasia or dysplasia was slightly noted on the noncancerous mucosa of the bile duct or gallbladder, differing from typical CBDD. However, the concept of cancer stem cells is widely accepted as important in cancer development, with recent studies showing that cancer stem cells play important roles in the carcinogenesis of various types of cancer. Several investigations have demonstrated that although the different components of mixed neuroendocrine-non-neuroendocrine neoplasms are monoclonal, their molecular signature is not identical to that of their relative counterparts when they present as separate neoplasms [12, 13].

In our case, it was probable that the adenocarcinoma first developed in the common bile duct with subsequent neuroendocrine differentiation. In contrast, the two previous reports of NEC-associated CBDD did not clearly indicate whether their tumor included adenocarcinoma components; thus, NEC may have developed de novo in the common bile duct.

One study reported that the incidence of cholangiocarcinoma was approximately 15 % in Japanese patients with CBDD [2, 3]. Another study reported that the overall incidence of cholangiocarcinoma with pancreaticobiliary maljunction, with both a dilated and a nondilated bile duct, is 10.4 %, which is more than 285 times higher than the risk in the general population [10]. The average age of patients with cholangiocarcinoma associated with pancreaticobiliary maljunction is 10 years lower than that of the general population [2, 14, 15].

Biliary NEC represents 0.19 % of all primary malignant tumors in the extrahepatic bile duct [16]. It is estimated that over 50 % of patients with gastroenteropancreatic NEC exhibit distant metastasis during initial diagnosis. Biliary NEC is relatively aggressive. In a review of 22 patients with biliary NEC, distant metastasis occurred in 16 cases on initial admission. Distant metastasis is present in all cases within one year after surgery, even though surgery is the mainstay of treatment for biliary NEC and is regarded as the curative option. The survival outcomes are poorer (in the decreasing order) for NECs that originate from the lungs, gastrointestinal tracts, and hepato-biliary-pancreatic systems [17]. Terashima et al., reported that the response rate to chemotherapy in patients with extrapulmonary NEC was lower than that in patients with pulmonary NEC. In cases of extrapulmonary NEC, the hepato-biliary-pancreatic systems group showed the lowest response rate [18]. In this case, preoperative imaging showed no distant metastasis, and the resection margin was negative. Furthermore, the disease has not recurred in this patient for over 16 months after surgery, which implies that this case is rare and valuable. Additionally, considering that distant metastasis within one year after surgery has been previously reported, adjuvant chemotherapy is important to prevent tumor recurrence. Recently, several investigations have reported the efficacy of immunotherapy for neuroendocrine neoplasms, which might become a novel therapy for advanced neuroendocrine tumors [19–25].

We present a rare case of a poorly differentiated NEC of the common bile duct associated with CBDD. Although the association between CBDD and NEC is unclear, CBDD is a known risk factor for carcinogenesis. As mentioned above, chronic inflammation results in adenocarcinoma, which transforms into neuroendocrine NECs. Thus, NEC should be considered a differential diagnosis for patients with cholangial tumors associated with CBDD. Although NECs generally have poor prognoses, no recurrence lesion was observed after surgery in this case, and the patient had a good course. Novel drugs, such as molecular-targeted drugs and immune checkpoint inhibitors, have been used for cancer; in the future, patients with biliary NEC might have a good prognosis, as in this case. To establish a more effective treatment, it is necessary to continue to accumulate similar reports.

## Data Availability

Not applicable.

## Abbreviations

CBDD: Congenital bile duct dilatation
NEC: Neuroendocrine carcinoma
